# Increased Power in Sediment Microbial Fuel Cell: Facilitated Mass Transfer *via* a Water-Layer Anode Embedded in Sediment

**DOI:** 10.1371/journal.pone.0145430

**Published:** 2015-12-29

**Authors:** Yoo Seok Lee, Junyeong An, Bongkyu Kim, HyunJun Park, Jisu Kim, In Seop Chang

**Affiliations:** School of Environmental Science and Engineering, Gwangju Institute of Science and Technology (GIST), 261 Cheomdan-gwagiro, Buk-gu, Gwangju, 500-712, Republic of Korea; Texas A&M University, UNITED STATES

## Abstract

We report a methodology for enhancing the mass transfer at the anode electrode of sediment microbial fuel cells (SMFCs), by employing a fabric baffle to create a separate water-layer for installing the anode electrode in sediment. The maximum power in an SMFC with the anode installed in the separate water-layer (SMFC-wFB) was improved by factor of 6.6 compared to an SMFC having the anode embedded in the sediment (SMFC-woFB). The maximum current density in the SMFC-wFB was also 3.9 times higher (220.46 mA/m^2^) than for the SMFC-woFB. We found that the increased performance in the SMFC-wFB was due to the improved mass transfer rate of organic matter obtained by employing the water-layer during anode installation in the sediment layer. Acetate injection tests revealed that the SMFC-wFB could be applied to natural water bodies in which there is frequent organic contamination, based on the acetate flux from the cathode to the anode.

## Introduction

Sediment microbial fuel cells (SMFCs) are being considered for use as a power source for aquatic water quality sensors such as pH, temperature, and dissolved oxygen sensors [1, 2, 3]. The greatest benefit in using SMFCs is that they utilize organic matter that is distributed in a natural aquatic environment as the fuel source for generating electricity [4, 5, 6]. Typically, for SMFC installation, the cathode electrode is exposed to the oxygen-rich aqueous phase, and the anode electrode is embedded in the organic-rich sediment without a membrane [7, 8, 9]. Electricity can then be produced based on the electron production from electrochemically active bacteria (EAB) or sulfate-reducing bacteria that use organic matter in the sediment as the electron donor [10]. However, it has been reported that the organic content in sediment is as low as 0.4% to 2.2% [11] and thus the anodes in SMFCs likely suffer from mass transfer limitations [12].

To overcome this inherent constraint, Rezaei et al. (2007) proposed the use of chitin and cellulose as an assistant substrate; this approach improved the power density in SMFCs by approximately 40 times, though further study is required regarding the installation and fabrication of anode electrodes before this approach becomes practical [13]. Shantaram et al. (2005) previously demonstrated the potential for utilising a manganese alloy as an electron donor, a so-called sacrificial anode, which led to a significant increase in the power output [14]. However, there is controversy over the classification of this abiotic anodic reaction-based fuel cell, which includes corrosion of the manganese alloy, as a microbial fuel cell. In more recent attempts to enhance the redox reactions of sulphate and sulphide, an intriguing concept for reforming the anode using anthraquinone-1,6-disulfonic acid was suggested by Lowy et al. (2008); they confirmed that a power density of 100 mW/m^2^ to 110 mW/m^2^ can be achieved, but the durability of the functional group on the anode electrode was not ensured [15, 16]. Furthermore An et al. (2013) proposed a simpler and easier approach for utilising microbial physiological characteristics to enhance both the current density and working voltage for a single SMFC [17, 18]. In their study, a maximum power density of 14.5 mW/m^2^ was observed at a sediment depth of 10 cm, which was 2.2 times higher than could be obtained at a sediment depth of 2 cm. However, this method requires the use of preliminary tests to determine the optimal anode depth for the SMFCs.

The mass transport of dissolved organics in sediment generally occurs by diffusion, with the diffusion rate of dissolved organics in sediment being much slower than in water phase. The physicochemical properties of the sediment that decrease the mass transport rate of dissolved organics include porosity, tortuosity, pore size, etc. Nonetheless, the effect of organic transfer rate in sediment on the performance of SMFCs has yet to be reported in literature. We believe that employing a separate water-layer during installation of the SMFC anode could facilitate the organic flux to the anode electrode, and subsequently improve the power output by increasing the anode kinetic activity of the MFCs. In this work, we demonstrate an “electrode-spacing method” which is to create a separate water layer surrounding the anode electrodes by using a fabric baffle inside the sediment, to enhance the organic transfer rate at the anode electrode. We also investigate the behavior of organic transfer in two different phases, sediment and water phase. We find that the water-layered anode structure for SMFCs (SMFC-wFB) was remarkably effective in terms of power and current increases, such that it could be an efficient way to facilitate the substrate transfer to an anode embedded in sediment, leading to an increase in the overall power output in SMFCs.

## Materials and Methods

### Electrode preparation

Rigid graphite plates (1 cm thickness, 4 cm length, 4 cm width) were used as the anode for the SMFC, while much larger graphite felt electrodes (2.54 cm thickness, 10 cm length, 10 cm width) were employed as a cathode to avoid cathodic limitations in the oxygen reduction reaction (ORR). A platinum wire (current collector, 0.7 mm diameter, 2 cm length) and a copper wire were twisted together and sealed with shrink tube. This wire was then inserted into a hole in the edge of the graphite plates, and sealed with poly epoxy to make it waterproof; the electrical resistances of the connections were negligible at 1 Ω. For the cathode electrode, titanium wire was used as the current collector instead of platinum wire.

### Installation and operation of two different types of SMFCs

**Fig 1:**
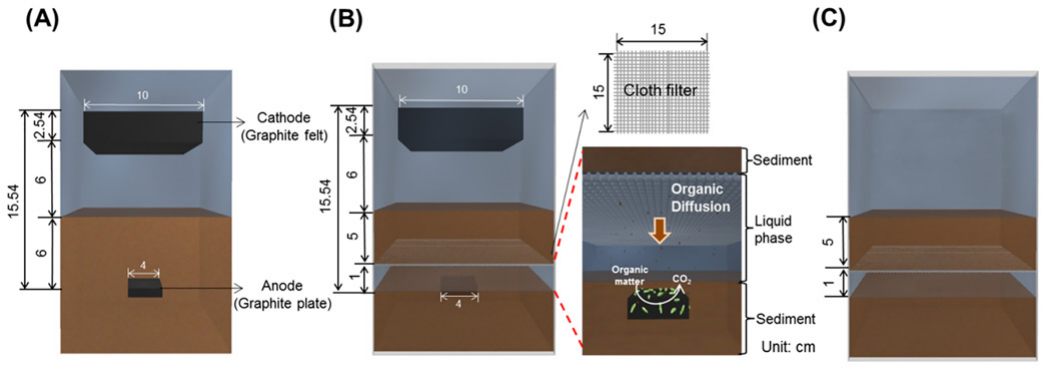
Configuration and dimensions for SMFC-woFB (A), SMFC-wFB (B) and SMFC-FB (C) installed in the MFC operating system.

To confirm data reproducibility, we used two different sediments and waters samples that were collected from two different local reservoirs (35° 21′ 38.47 N, 126° 25′ 25.22 E; 35° 11′ 27.6”N, 126° 47′ 53.4 E). The sampling of sediments and waters in the reservoirs was conducted in accordance with the laws of South Korea; the two local reservoirs for the sampling of waters and sediments were not privately-owned or protected areas, and were not involved with endangered or protected species; no specific permissions were required for the sampling of sediments and waters in the two reservoirs (i.e., 35° 21′ 38.47 N, 126° 25′ 25.22 E; 35° 11′ 27.6”N, 126° 47′ 53.4 E). Once collecting the sediment and water samples from the reservoirs, we immediately moved the samples to a laboratory for SMFC set-up and analyses. One of the sediment samples contained acetate over 5 mM, whereas there was no acetate in the other sediment, designated as sediment-wAc and sediment-woAc, respectively. The water samples from the two different reservoirs contained no acetate, and a measured COD of nearly 0. The sediment samples were physically mixed in a laboratory for 2 h to ensure that the organic and inorganic contents of the sediment were completely homogenized prior to use. The installation of an SMFC without fabric baffle (SMFC-woFB) consisted of filling acrylic aquariums (15 cm × 15 cm × 30 cm) with 8 cm of sediment-wAc and sediment-woAc (see Fig 1A). Thereafter, the prepared anode was buried at a depth of 6 cm into the paved sediment samples so that they were ~2cm from the bottom. Then, 10 cm of reservoir water, collected from the different reservoirs, was added to each aquarium. Finally, a cathode electrode was positioned 6 cm above the sediment surface in the waster phase (see Fig 1A).

The SMFC with a fabric baffle (mesh filter cloth, Kavon Filter Products Co.) was constructed using 10–100 μm pore size mesh (denoted as SMFC-wFB). We then added sediment-wAc and sediment-woAc into identical aquariums as those constructed for the SMFC-woFB; paved at a 2 cm height from the bottom, with the anode placed widthwise on the sediment surface layer of the aquariums (see Fig 1B). Next, 2250 mL of the collected reservoir water was filled into each aquarium, to a 1 cm water level from the sediment surface of the aquariums (see Fig 1B). After that, the fabric baffle was placed such that it covered the water surface (referred to as the water-layer) and fixed with clips to maintain a 1 cm of water layer between the baffle and the sediment surface of the aquariums (see Fig 1B). Finally, a 5 cm sediment layer was constructed on the fixed fabric baffle (see Fig 1B), and reservoir water was subsequently added to the aquarium to a water level of 10 cm (DO: 5.12 ± 0.23 mg/L). The cathode of SMFC-wFB was positioned 6 cm above the top sediment layer surface, at an identical height to the cathode of SMFC-woFB. Installed with sediment-woAc, SMFC-wFB and -woFB were used to confirm data repeatability and for acetate injection tests (see the following section for a description of the tests).

### Acetate Injection Tests

Sodium acetate (c.a., 30 mM, 20 mL) was injected with a 50 mL of syringe into the catholyte of the SMFC-wFB-woAc and SMFC-woFB-woAc in order to compare the mass transfer rates of the organic matter from sediment/water boundary to the surface of the anode electrode. On injection of the acetate, the catholyte (i.e., reservoir water in the aquariums) was mildly agitated for 5 min using an impeller at 20 rpm. We then monitored the evolution of the current and electrode potentials of the both SMFC systems with respect to time; the acetate concentration of the sediment water layer in SMFC-wFB was concurrently monitored every 1 h. Note that the initial acetate concentration of the sediment-woAc and its reservoir water was lower than detection limit of HPLC. To estimate acetate transfer rate from the sediment surface to the separate water-layer, 30 mM of sodium acetate was directly injected into the sediment-woAc by using a needle in which only a fabric baffle was installed without an anode and cathode electrode (see Fig 1C for details). Then, acetate concentration in the water layer was periodically measured.

### Analyses

The open circuit voltages (OCV), closed circuit voltages (CCV), and anode electrode potentials of the MFCs were monitored and recorded using a data acquisition system (Multimeter 2700, Keithley Co., Cleveland, OH). The anode potentials were measured using Ag/AgCl reference electrodes (MF-2052; Bioanalytical Systems Inc., West Lafayette, IN) that were placed as close as possible to the cathode electrodes (around 0.2 cm). During each measurement, the time interval for data acquisition was varied from 10 s to 30 min depending on the experiment being performed [10].

The voltage-current (I-V) curves of the SMFCs were drawn using the data obtained from the polarization tests for the SMFCs. Polarization tests were performed using variable (external) resistances that ranged from 300 k ances that The external resistances were sequentially switched from high (100 kΩ) to low (500 Ω); each resistance was applied for at least 15 min until the current for each resistor became stable.

Internal resistances including solution resistance were estimated via electrochemical impedance spectroscopy (EIS) using an Autolab Potentiostat equipped with an FRA-ADC impedance module (PGSTAT302, Eco Chemie, Utrecht) under a frequency range of 100 kHz to 0.01 Hz. The onset voltages (two-electrode configuration: working and counter electrode) were OCVs having a ± mV amplitude [10].

Conductivity was measured using a 3-star desktop conductivity meter (Thermo Fisher Scientific Inc., Waltham, MA); the conductivity the catholyte ranged from 507 to 540 *μ*S/cm. The dissolved oxygen concentration and pH were measured using a 4-star DO pH meter (Thermo Fisher Scientific Inc.). Dissolved oxygen and pH in the catholyte ranged from 7.21 to 7.65 mg/L and 7.52 to 7.67 during our experiments. The acetate concentration of the water phase in the anode region was measured using a high performance liquid chromatography (HPLC) system (Waters Assoc., Milford, MA).

The content of organic material in the sediment (as volatile solids (VS)) was analyzed by drying the sediment (105°C, 48 h) and then combusting the dried sample (550°C, 5 h) [18, 19, 20]; the organic content of the sediment was 5.2 ± 0.7% (w/w). The concentration of organic content in liquid phase (as soluble organics) was analyzed by converting the chemical oxygen demand (COD) to equivalent organic carbon (as CH_2_O equivalent) [21].

The soluble chemical oxygen demand (SCOD) of the catholyte or anolyte of SMFC-wFB was measured using a COD analysis system (HS-COD-LR&MR, Humas Co. Ltd., Korea); the organic concentration (as CH_2_O equivalent) of the anolyte of SMFC-wFB was 19.66 ± 2.37 g/L.

## Results and Discussion

### Higher performance in SMFC-wFB than -woFB

**Fig 2:**
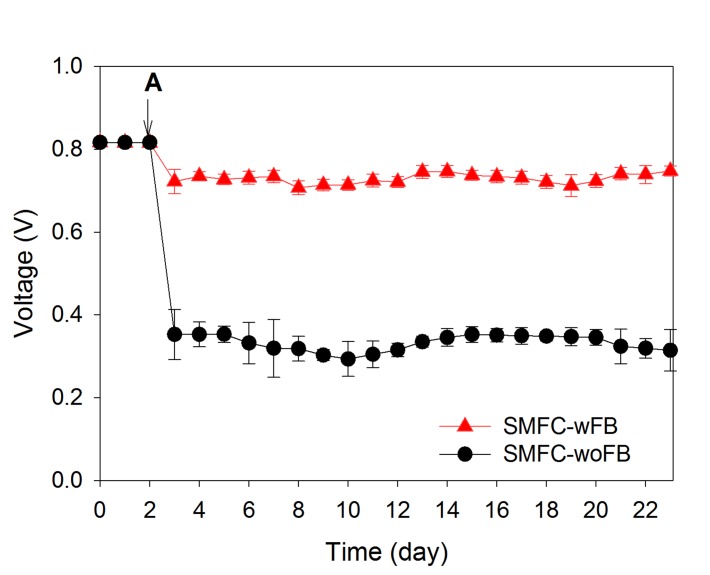
Voltage measurements of SMFC-woFB and SMFC-wFB under closed circuit mode at a 5 knd SMFC-wFBls CHas CHteas analyzed by conver The arrow indicates when the operation mode was changed to CCV.

Fig 2 presents the OCV and CCV developments in SMFC-wFB and-woFB which were installed with sediment-wAc (see Materials and Methods). At day 2, the OCV stabilized at 0.82 ± 0.02 V in SMFC-woFB and 0.81 ± 0.01 V in SMFC-wFB. After the MFCs were changed to closed circuit mode by employing a 5 kΩ external resistance, the current in the MFCs stabilized at 0.07 mA for 0.35 V (SMFC-woFB) and 0.14 mA for 0.72 V (SMFC-wFB) (Fig 2).

**Fig 3:**
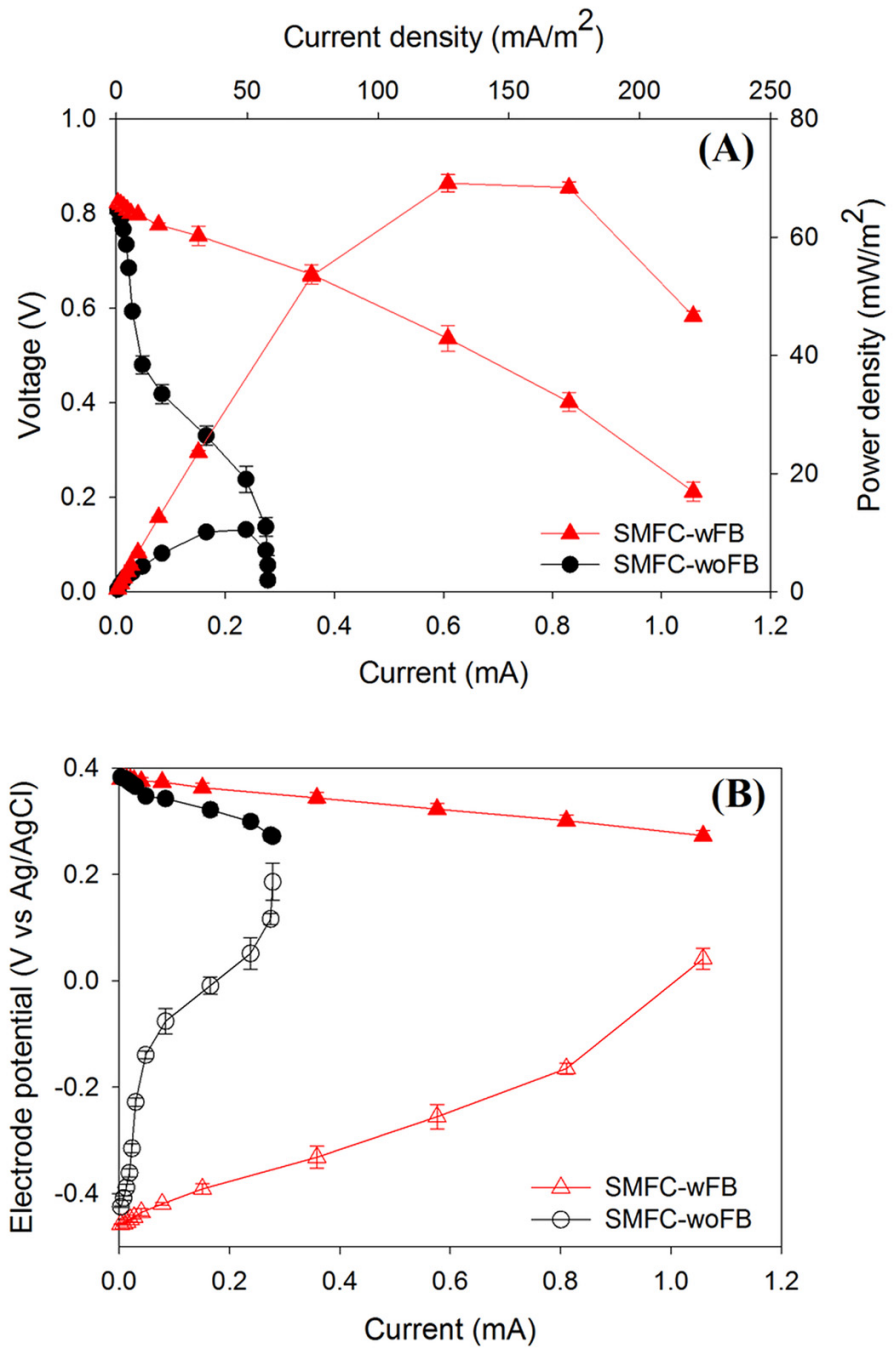
(A) Performance curves for the SMFC-woFB and SMFC-wFB installed with sediment-wAc; (B) electrode potential plots for anode and cathode monitored during the polarization tests for the MFCs.

To confirm whether or not the performance of SMFC-wFB was superior to SMFC-woFB through all external resistors, we performed polarization tests on the MFCs. As shown in Fig 3A, the maximum power and current density were higher in SMFC-wFB (69.1 mW/m^2^ for 220 mA/m^2^), as compared to those in SMFC-woFB (10.5 mW/m^2^ for 57.1 mA/m^2^). The overvoltage for the current of 0.2 mA was as small as 0.09 V in SMFC-wFB, whereas SMFC-woFB had high overvoltage of 0.53 V at the identical current (see Fig 3A). There could be two possible reasons for the different performances in SMFC-wFB and SMFC-woFB. First, it could be due to the difference in the internal resistance of the MFCs; and second, it could be due to the difference in the anode kinetics of the MFCs because all operational conditions for the cathode in the SMFCs were identical. As seen in Table 1, the ohmic resistance between the anode and cathode of SMFC-woFB was observed to be 207 ± 0.7 Ω whereas the resistance for SMFC-wFB was slightly higher at 228 ± 0.1 ΩwaThe ohmic resistance between the cathode and top sediment layer surface in the MFCs was similar at 35.8 Ω. The 1 cm water layer in the middle of the sediment could be responsible for the slightly higher ohmic resistance observed in the SMFC-wFB (see Fig 1). The higher ohmic resistance in SMFC-wFB does not support the argument that the higher performance in SMFC-wFB might be due to lower ohimc resistance in SMFC-wFB than in -woFB.

**Table 1:** Internal resistances of SMFCs obtained from electrochemical impedance spectroscopy: internal resistances between the water-sediment interface and anodes and between the cathodes and the water-sediment interface.

	Internal resistance (Ω)		
	Water-sediment interface to anode	Cathode to water-sediment interface	Total internal resistance
SMFC-woFB	171 ± 0.3	35.8 ± 0.03	207 ± 0.7
SMFC-wFB	192 ± 0.7	35.8 ± 0.01	228 ± 0.1

To investigate whether the better performance in SMFC-wFB was due to the higher anodic kinetic activity, the evolution of electrode potentials in SMFC-wFB and -woFB were monitored during the polarization tests for the MFCs (see Fig 3B). In the figure, the anode and cathode open circuit potentials of the MFCs were similar at -0.45 and 0.37 V, respectively; the anode and cathode overpotential in the both types of SMFCs then increased by lowering the external resistors. The anode overpotential at the current of ~0.2 mA in SMFC-woFB was substantial at ~-0.48 V, whereas SMFC-wFB had small anode overpotential of -0.09 V at the same current. In comparison, the cathode overpotential in SMFC-wFB was slightly smaller at -0.02 V than the anode overpotential in SMFC-wFB, and the cathode overpotential of SMFC-woFB was much smaller at -0.09V compared to the anode overpotential in the MFC. It was observed that 90.7% of the total overvoltage for the current of 0.2 mA in SMFC-woFB was responsible for the anode overpotential of 0.48 V. These results clearly indicate that the anode kinetic activity in SMFC-wFB was much more facile compared to that in SMFC-woFB.

The initial acetate concentration of the reservoir water used to create the water layer for the SMFC-wFB was lower than the detection limit of HPLC (see Materials and Methods), but after 2 days of operation the liquid sample collected from the same water layer had a 3.1 mM acetate concentration. It is posited here that acetate is being eluted from the sediment through the fabric baffle. However, the COD for 3.1 mM acetate (5.5 mg COD / g liquid) is 10.7 times lower compared to that contained in the sediment (57 mg / g sediment), which means that though SMFC-woFB had more substrate it did not produce a higher performance than SMFC-wFB. The higher performance in the SMFC-wFB, which had a much lower COD, implies that there was a much more facile mass transfer in the water layer than that in the sediment layer. To confirm this interpretation, we injected acetate in the cathode solution and monitored the current evolution of both SMFC-woFB and -wFB installed in the sediment-woAc (see Materials & Methods for more details). The diverse organics such as acetate, propionate, lactate, pyruvate and butyrate in sediment is an electron source for electrochemically active bacterial (EAB) or sulfate-reducing bacteria (SRB) which are directly or indirectly involved in electricity generation of SMFCs [18, 22, 23]. These bacteria are widely distributed in most sedimentary environments [24, 25, 26]. For this reason, we believed that EAB (or SRB) could oxidize the acetate that was added to sediment-woAc, which was discussed in the following section.

### Substrate diffusion rate in SMFC-woFB and -wFB

**Fig 4:**
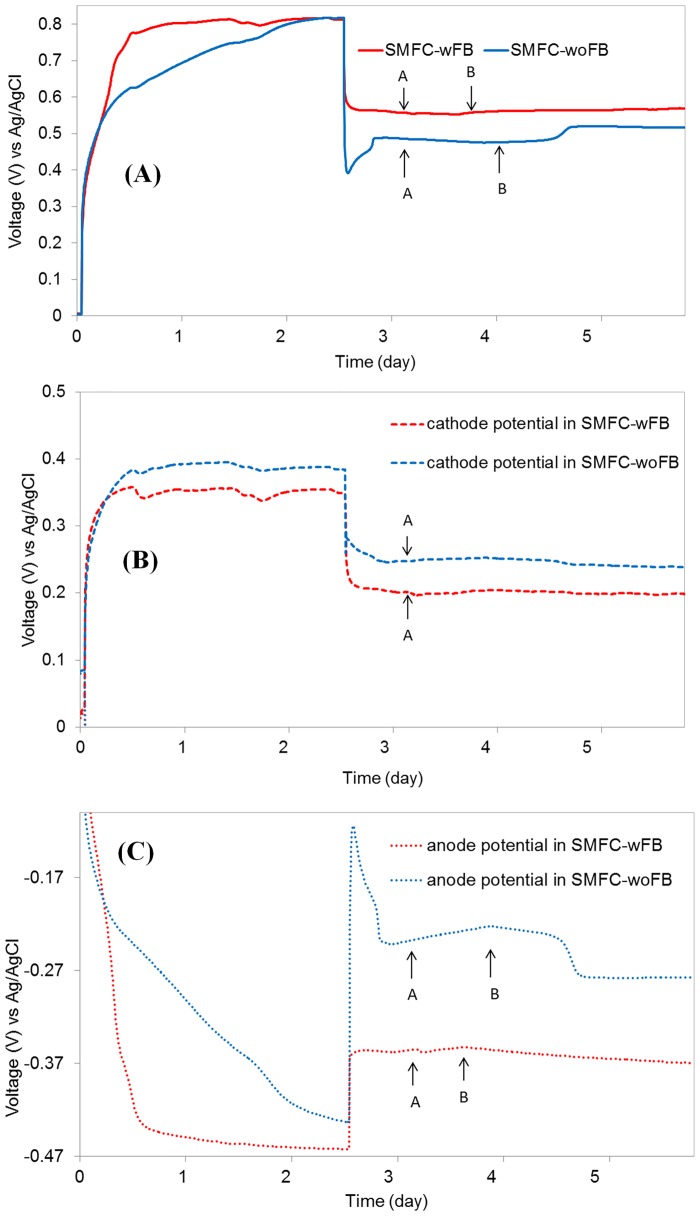
Aceate injection tests for the SMFC-woFB and SMFC-wFB constructed with sediement-woAc; OCV and CCV evolution (2 kΩ) in the MFCs (A), development of cathode potential (B) and anode potential (C) in the MFCs; the arrows with the letters “A” and “B” mark the acetate injection points and the resulting current response points respectively.

Fig 4A presents the evolution of OCV and CCV (at 2 kΩ) in SMFC-wFB and -woFB after the injection of acetate (30mM) into the catholyte. The currents in SMFC-wFB and -woFB stabilized at 0.28 mA and 0.24 mA, respectively, similar to the initial current after a couple of hours. However, after 12 h, the current in SMFC-wFB gradually increased and then was saturated at 0.296 mA at 6 h prior to that of SMFC-woFB. In comparison, the current in SMFC-woFB started increasing 2 h later than that of SMFC-wFB and became saturated at 0.261 mA after 8h from the acetate injection. As presented in Fig 4, during the increase in the current of the MFCs the anode potential decreased from -0.35 V to -0.38 V in SMFC-wFB and -0.22 V to -0.30 V in SMFC-woFB. In contrast, the change of the cathode potential (cathode overpotential) in SMFC-wFB and -woFB was relatively negligible at 0.21 V and 0.23 V. From these results, it is clear that the acetate in SMFC-wFB reached the anode earlier than that of SMFC-woFB, indicating that the acetate diffusion rate in the 1 cm of the water layer for the anode of the SMFC-wFB was much more facile when compared to an identical thickness of sediment layer (i.e., 1 cm).

**Fig 5:**
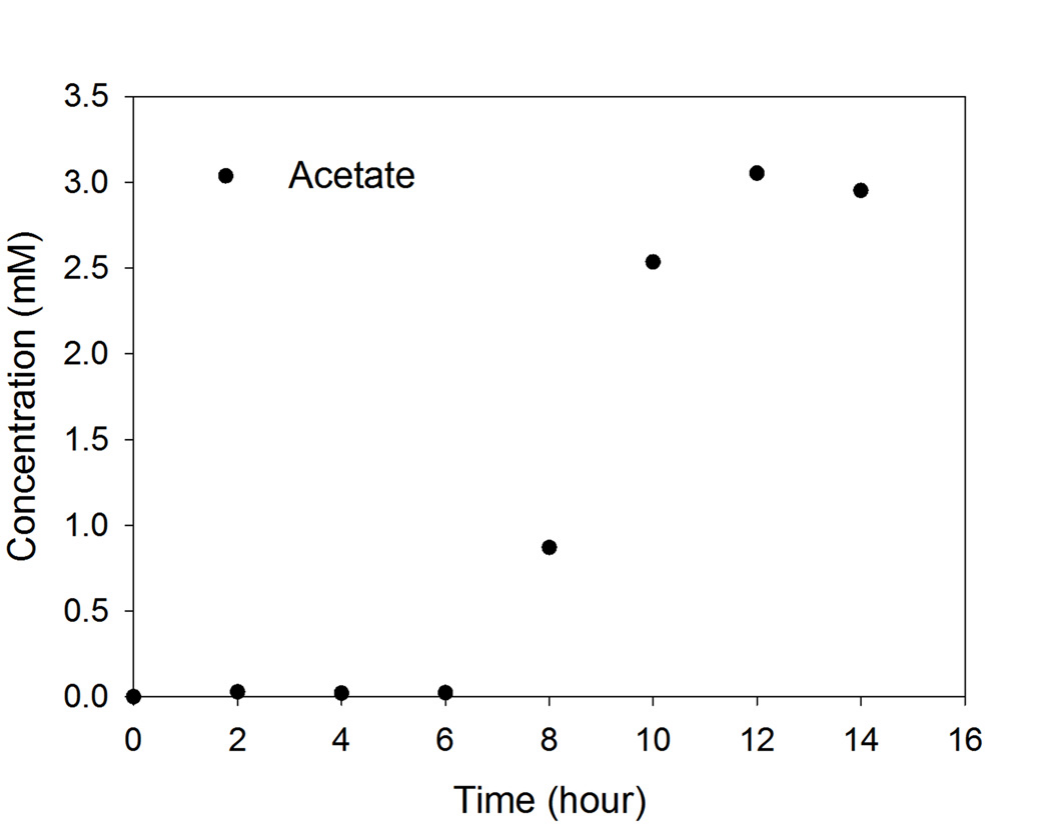
Change of acetate concentration in the water-layer after 30 mM acetate injection to the cathode of the SMFC-FB installed with sediment-woAc.

However, the current increment of SMFC-wFB by the acetate injection to the cathode could be considered to be negligible. To access the time taken for acetate diffusion from the cathode to the water layer, we performed a separated acetate injection test using sediment and a fabric baffle with no anode and cathode electrode (see Fig 1C for experimental details). The initial acetate concentration of the water layer was lower than the detection limit of HPLC even after a couple of hours operation (Fig 5). Six hours after acetate injection to the center of the sediment-FB the acetate concentration in the water layer between the fabric baffle and the lower sediment started increasing, and then was saturated at 3 mM in another 6 h (Figs 1C and 5). From this result, it can be found that the current response to the acetate injection, shown in Fig 4, was 6h later than acetate arrival to the water layer, suspecting that acetate diffusion rate via the 1 cm of the water layer in SMFC-wFB could be very slow when considering current response time. However, the late current response at the earlier acetate arrival to the water layer could be attributed to number of EAB activity or their population density on the anode electrode.

**Fig 6:**
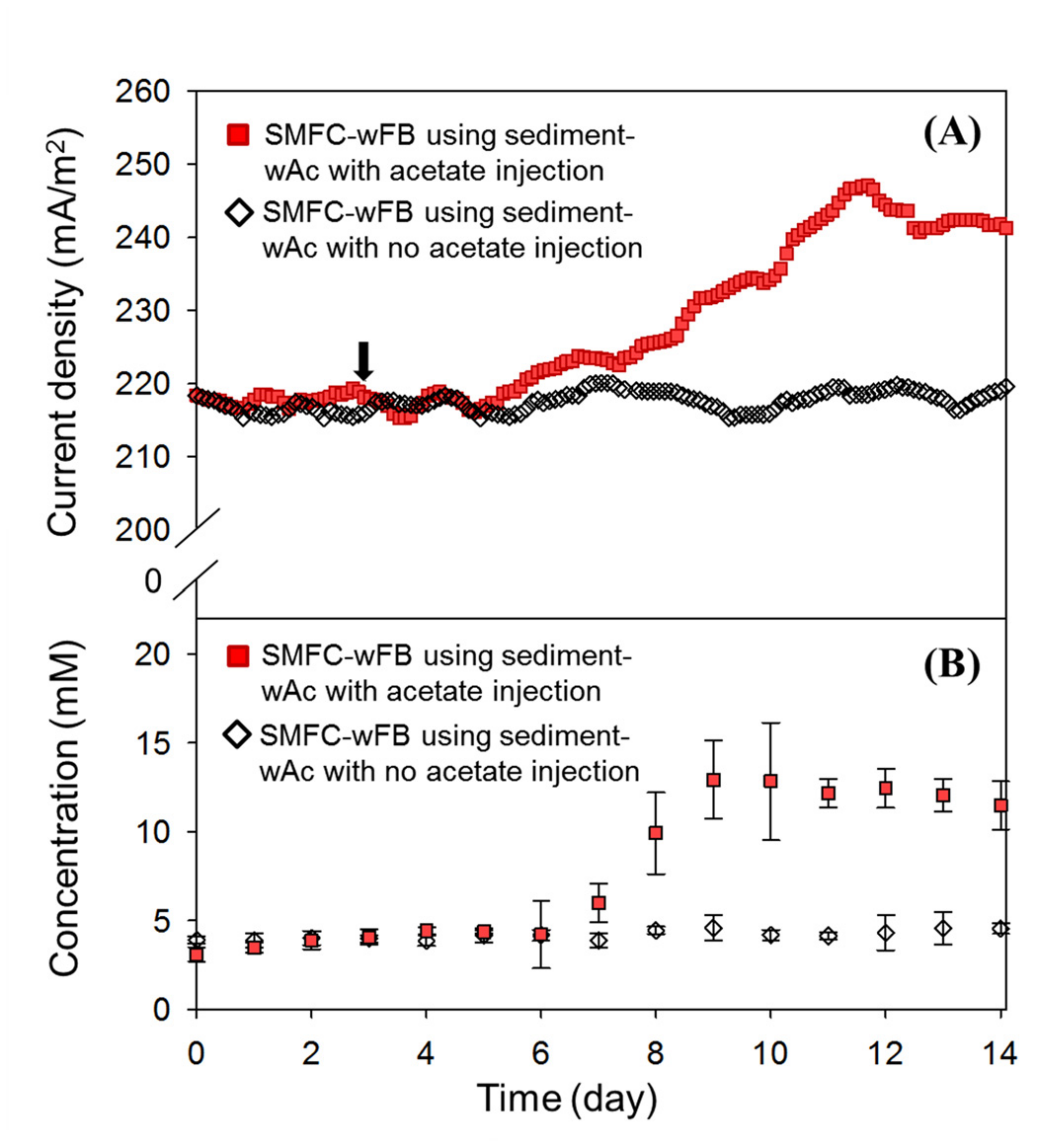
Change of current density and acetate concentration on SMFC-FB by injection of acetate. Arrow indicates acetate injection into the catholyte at the water-sediment interface in SMFC-wFB. No acetate injected SMFC-wFB was also operated as a control set.

To investigate the acetate diffusion rate, SMFC-wFB installed in sediment-woAc (but not SMFC-wFB constructed with sediment-wAc) was used for acetate injection test. Accordingly, we suspected the most EAB on the anode of the SMFC-wFB, installed in the sediment-woAc, might be not favorable to acetate utilization [23]. It is well known that some EAB such as *Geobacter* spp. can use acetate as an electron donor [15, 16]. Thus, it was believed that a sulfide-sulfur-sulfate cycle [27, 28] which is another pathway for electricity generation of SMFCs could be involved in the anode of SMFC-wFB installed in the sediment-woAc. Sulfate reducing bacteria (SRB) and sulfate-oxidizing bacteria (SOB) have a key role in the cycle; sulfides from SRB donate electrons to electrodes and are oxidized to elemental sulfur on the anode electrode, and then are further oxidized into sulfate by SOB. These interpretations stated above could be supported with the distinct increase in the current by the acetate injection to the SMFC-wFB installed in the sediment-wAc (see Fig 6).

As seen in Fig 6, on injection of 30 mM acetate to the catholyte of the SMFC-wFB installed in the sediment-wAc the current significantly increased from 1.05 to 1.19 mA, 10 times higher than the SMFC-wFB constructed in sediment-woAc (also see Fig 4A). The small and sluggish current response to the acetate injection in the SMFC-wFB installed in the sediment-woAc could be due to a small population of EAB that directly use acetate. These results support the hypothesis that the organic transfer rate via the separate water layer in the SMFC-wFB is much more facile compared to the continuous sediment layer in the—woFB analogue.

**Fig 7:**
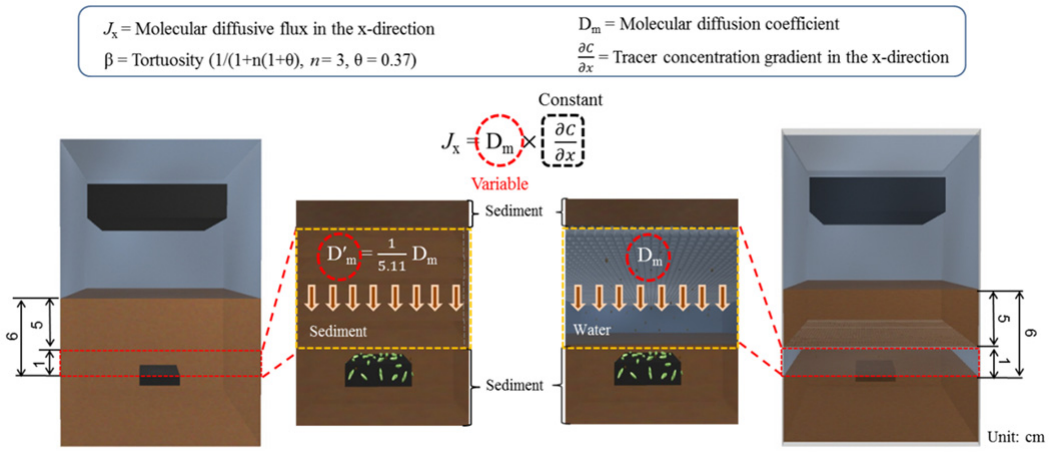
Physical explanations of difference in the molecular diffusion rate in water and sediment-layer on the anode described by Fick’s first law.

Finally, to propose the physical explanation about how the water-layer makes the difference in the anode kinetic, Fig 7 was added that molecular diffusion is described by Fick's first law, which explained that solute movement rate is proportional to the spatial concentration gradient, given, in one dimension, by

$${\text{J}}_{\text{x}}=\;-{\text{D}}_{\text{m }}\times \left(\delta \text{C}/\delta \text{x}\right)$$ (1)

where: J_x_ is the molecular diffusive flux in the x-direction, D_m_ is the molecular diffusion coefficient and ∂C/∂x is the tracer concentration gradient in the x-direction. In case of solutes in water, molecular diffusion value typically range between 0.5 to 2.0 /10^9^ · m^2^/s and are determined empirically [29]. However, the coefficient of molecular diffusion through sediment is different from that through free fluid. The solute must flow around the sediment particles, creating a longer flow path and thereby effectively reducing the coefficient. The molecular diffusion coeffcient through sediment (D′_m_), has been explored theoretically [30], as well as empirically by relating it to sediment tortuosity that is related to sediment porosity (θ), by several studies [31]. This leads to the general expression for molecular diffusion in sediments

$${\text{D}\prime }_{\text{m }}=\beta \times {\text{D}}_{\text{m }}$$ (2)

where: β represents an empirical expression for tortuosity as a function of sediment porosity and is described by Iversen and Jrgenson [32] as

β=1/(1+n(1+θ)) (3)

where: n is the sediment type constant (n = 3 for clay-silts and n = 2 for sands). However, n = 3 was used by O'Connor and Harvey [33] throughout their analysis, regardless of sediment type. The sediment porosities (θ) measured are slightly higher than the theoretical value of 0.37 for randomly placed spheres [34]. Therefore, diffusion coefficient of material around anode area in SMFC-woFB and—wFB could be only one important parameter that determines the substrates transfer rate to anode because other parameters for molecular diffusive flux (i.e. sediment tortuosity, tracer concentration gradient) were same due to all operational conditions in the SMFCs were identical; as a results, we could expect SMFC-wFB which has 1 + *n*(1 + θ) times higher diffusion coefficient than SMFC-woFB has enhanced mass transfer rate for kinetically favorable tor anodphiles and electrophiles.

## Conclusion

The maximum current density (220.46 mA/m^2^) and maximum power density (69.14 mW/m^2^) of an SMFC having a separate water layer (SMFC-wFB) in the anode sediment were 3.9 and 6.6 times higher than the respective values for an SMFC having no water layer (SMFC-woFB). It was found that the organics transfer rate in the separate water layer was much more facile compare to that for the sediment layer; as a result, the anode kinetic activity in the water layer was enhanced and the performance in the SMFC-wFB substantially increased. It is expected that this water layer in the anode structure for SMFCs can be an efficient way to facilitate the substrate transfer to an anode embedded in sediment, leading to further increases in the power output in SMFCs.
